# Costly defense in a fluctuating environment—sensitivity of annual *Nothobranchius* fishes to predator kairomones

**DOI:** 10.1002/ece3.3019

**Published:** 2017-05-07

**Authors:** Matej Polačik, Michal Janáč

**Affiliations:** ^1^ Institute of Vertebrate BiologyBrnoCzech Republic

**Keywords:** *Clarias*, diapause, embryo, olfactory cue, tilapia

## Abstract

Antipredator strategies increase the chances of survival of prey species but are subject to trade‐offs and always come at a cost, one specific category being the “missed opportunity.” Some animals that can modulate the timing of life‐cycle events can also desynchronize this timing with the occurrence of a predator. In an unpredictable environment, such a modification may result in a mismatch with prevailing conditions, consequently leading to reproductive failure. In eastern Africa, temporary pools existing only during the rainy season are inhabited by annual fish of the genus *Nothobranchius*. We examined (i) the capability of multiple *Nothobranchius* populations and species to cease hatching when exposed to chemical cues from native fish predators and adult conspecifics and (ii) the ability of *N. furzeri* to modulate their growth rate in the presence of a gape‐limited fish predator. As the tested *Nothobranchius* spp. originate from regions with extreme environmental fluctuations where the cost of a missed opportunity can be serious, we predicted an inability to cease hatching as well as lack of growth acceleration as both the predator's gape limitation and the environment select for the same adaptation. Our results showed no biologically relevant influence of kairomone on hatching and no influence on growth rate. This suggests that, in an unpredictable environment, the costs of a missed opportunity are substantial enough to prevent the evolution of some antipredator defense strategies.

## 1 Introduction

Animal species that are likely to be preyed upon during their lives have evolved countless antipredator strategies to increase their chances of survival. Examples include altering their behavior (Apfelbach, Blanchard, Blanchard, Hayes, & McGregor, 2005), morphology (Bourdeau & Johansson, 2012; Lass & Spaak, 2003), or life‐history strategy (Weider & Pijanowska, 1993). All predator‐induced countermeasures are costly and subject to trade‐offs (Dayton & Fitzgerald, 2011; Frommen et al., 2011; Lima & Dill, 1990; Smith & Webster, 2015). If they were not, they would have been favored by natural selection and become standard phenotypes. Typically, an antipredator strategy decreases overall energy intake (Bourdeau, Bach, & Peacor, 2016; Hall & Clark, 2016) or redirects available energy to defense (Scherer, Lunt, Draper, & Smee, 2016; Van Buskirk, 2000), which is often manifested as decreased fecundity (Brönmark et al., 2012). Costs may also be less straightforward, such as a higher vulnerability to a pathogen due to decreased investment into immunity (Yin, Laforsch, Lohr, & Wolinska, 2011).

Another common trade‐off category is that entailing “opportunity costs,” where a predator is avoided at the expense of a missed opportunity, for example loss of energetic gain through an omitted feeding event (Smith & Webster, 2015). Usually, the “currency of payment” is the time spent in a hideout or length of time spent in a dormant stage or as a juvenile, which diminishes the efficiency of foraging, mate choice, or reproduction (Reaney, 2007). Conflict between the decision to invest or not invest time into an antipredator measure is most prominent in unpredictable environments where there is an additional threat of catastrophic, condition‐independent mortality. The costs of a missed opportunity, which would only result in a relatively low decrease in fitness in a stable environment, may have much more serious consequences when combined with the imminent risk of a catastrophic event wiping out the entire population. Delayed onset of reproduction resulting from alteration of a life‐history strategy due to the presence of a predator, for example, could entail complete reproductive failure if the catastrophe comes before first reproduction (Dayton & Fitzgerald, 2011). Missed opportunity costs play a major role under stochastic conditions and are likely to rival predation risk in terms of relative importance to prey fitness.

Temporary pools are diverse ecosystems, yet their stochastic nature presents a continuous risk of desiccation for water organisms. Despite the harsh conditions, several groups of fish have successfully colonized these habitats. In Africa, annual killifish of the genus *Nothobranchius* are completely adapted to long‐term survival in such pools, surviving through the dry season as eggs encased in the dry mud. When the pools fill again following the seasonal rains, the fish hatch, grow, and mature extremely quickly in order to reproduce as soon as possible (Blažek, Polačik, & Reichard, 2013), thereby producing the next generation before the pool once again dries out. *Nothobranchius* fishes must cope with the risk of predation and desiccation simultaneously. Their fish predators include lungfish *Protopterus annectens Owen*, sharptooth catfish *Clarias gariepinus* Burchell, and tilapiine cichlids like *Oreochromis* sp. or *Coptodon sp*. (Pinceel et al., 2015; Reichard, 2010, 2015; Reichard, Polačik, & Sedláček, 2009). While catfish and tilapiines are not adapted to survive through prolonged dry phases, they occasionally colonize temporary pools during flood events. For example, our field data from Mozambique show that either a tilapiine, a catfish, or both were present in 22% of samples from the pools, at least in some years with a documented presence of *Nothobranchiu*s fishes. If the pool is invaded by predators before the *Nothobranchius* eggs start to hatch, the killifish embryos can potentially avoid catfish and tilapia predation by postponing hatching. By skipping the current inundation and hatching during a future inundation event during the same rainy season (Polačik et al., 2014), the fish predators will be wiped out by the dry phase in between (Pinceel et al., 2015). The physiological ability of *Nothobranchius* embryos to detect chemical cues from the surrounding environment has been confirmed, with Inglima, Perlmutter, and Markofsky (1981), for example, showing that embryos of *N. guentheri* Pfeffer are capable of halting early embryonic development in the presence of adult conspecifics.

Here, we test species‐ and population‐specific capability of *Nothobranchius* spp. to adjust life‐history strategies in the presence of predator‐released kairomone, a chemical substance released by a predator and used by prey to detect its presence in the environment (Ruther, Meiners, & Steidle, 2002). Our study expands upon that of Pinceel et al. (2015), who showed that eggs of Tanzanian *N. steinforti* Wildekamp did not hatch in water conditioned by kairomones from a nonnative, American pumpkinseed *Lepomis gibbosus* Linnaeus. Reaction to a kairomone may vary across close species (Pietrzak, Dawidowicz, Prędki, & Dańko, 2015; Van Dam & Walton, 2008; Walton, Van Dam, & Popko, 2009) as well as across populations (Dalesman, Thomas, & Rundle, 2015; Miyakawa, Sugimoto, Kohyama, Iguchi, & Miura, 2015; Walsh & Post, 2012), while different predators can induce different effects in a single prey species (Weber, 2003). In addition, the Tanzanian *N. steinforti* lives in a generally humid region with two rainy seasons per year and high rainfall reliability (Hamisi, 2013), where the costs of postponed hatching in terms of the missed opportunity are less likely to occur.

We tested the reaction of three *N. furzeri* Jubb populations and three *Nothobranchiu*s species (*N. furzeri*,* N. orthonotus* Ahl, and *N. pienaari* Shidlovskyi, Watters and Wildekamp) to kairomones released by adult conspecifics and two species of a native, coevolved predator in order to reveal whether the previously reported postponed hatching in the humid region occupying *N. steinforti* (Pinceel et al., 2015) can be regarded as a general rule for *Nothobranchius* fishes. In addition, we used a native, gape‐limited redbreast tilapia *Coptodon rendalii* Boulenger to reveal whether *N. furzeri* can modulate their growth rate (Chandler, Gorman, & Haas, 2016) in response to olfactory cues from the predator.

All the *Nothobranchius* species used live in southern Mozambique, at the southernmost margin of the genus’ distribution. Here, rainfall is highly erratic, and reliance on a future inundation event is a high‐risk strategy (Mazuze, 2007; Reichard, Polačik, Blažek, & Vrtílek, 2014) as postponed hatching may ultimately transform into total reproductive failure (Dayton & Fitzgerald, 2011). Hence, the selective pressure of the environment, which acts antagonistically to the predation pressure, is stronger than in the relatively more humid regions. With regard to potential costs, therefore, we predicted no reaction to kairomones in the hatching experiment. Similarly, reduced growth rate prolongs reaching sexual maturity and increases the risk of habitat desiccation. While some animals can decrease their encounter rate with a predator through slowing down their growth (Bjærke, Andersen, & Titelman, 2014), the gape limitation of tilapiines instead represents an achievable size refuge for killifish, facilitating acceleration in the growth rate. However, the unpredictable environmental conditions also strongly support tendency toward a fast growth (Blažek et al., 2013) and therefore act in concert with the pressure from the predator. We assumed that in the tested species *N. furzeri* the abiotic conditions have already resulted in the evolution of a maximum physiological growth rate and predicted that the presence of olfactory cues from *C. rendalli* will not result in any additional growth rate increase.

## 2 Materials and Methods

### 2.1 Hatching experiment

*Nothobranchius* eggs were obtained during a two‐day group spawning of 15–20 pairs from each of the three populations and the three test species into peat substrate (Polačik, Blažek, & Reichard, 2016). The clutches represented an F5 generation of wild‐caught fish imported from Mozambique in 2012 (for details on collecting codes and GPS coordinates, see Table 1). After incubating in the peat for 3 months at 25°C, eggs ready to hatch (recognized by their conspicuous golden eyes) were handpicked from the peat no more than 2–3 hr before the experiment in order to avoid desiccation (Polačik et al., 2016). In *Nothobranchius* spp., the eggs that reached their prehatching stage can delay their hatching even when provided with standard hatching stimuli (Pinceel et al., 2015).

**Table 1:** Final hatching rate (after 72 hr) and hatching pace (the proportion of eggs hatched after 12 hr to those hatched after 72 hr) of *Nothobranchius* spp. treated with a predator kairomone and control group

			Eggs			Hatching pace		Hatching rate	
Population/species	Method	Predator	12 hr	72 hr	N	%	*p*	%	*p*
*N. furzeri* MZCS 2	ex situ	control	32	34	38	94.1		89.5	
S24 03.808		adults	28	34	50	−11.7	.259	−21.5	**.021**
E32 43.932		catfish	NA	NA	NA	NA	NA	NA	NA
		tilapia	39	39	50	+5.9	.214	−11.5	.252
*N. furzeri* MZCS 222	ex situ	control	47	49	50	95.9		98.0	
S21 52.414		adults	46	50	50	−3.9	.678	+2.0	1.000
E32 48.039		catfish	40	44	50	−5.0	.417	−10.0	.112
		tilapia	43	48	50	−6.3	.268	−2.0	1.000
*N. furzeri* MZCS 414	ex situ	control	36	39	45	92.3		86.7	
S22 33.278		adults	47	48	50	+5.6	.321	+9.3	.144
E32 43.635		catfish	41	47	50	−5.1	.502	+7.3	.300
		tilapia	47	50	50	+1.7	1.000	+13.3	**.009**
*N. furzeri* MZCS 414	in situ	control	17	40	50	42.5		80.0	
S22 33.278		adults	22	38	50	+15.4	.257	−4.0	.807
E32 43.635		catfish	5	19	20	−16.2	.264 264	+15.0	.157
		tilapia	23	40	50	+15.0	.264	0.0	1.000
*N. orthonotus* MZCS 2	in situ	control	48	49	50	98.0		98.0	
S24 03.808		adults	43	45	50	−2.4	.605	−8.0	.204
E32 43.932		catfish	49	49	50	+2.0	1.000	0.0	1.000
		tilapia	48	50	50	−2.0	1.000	+2.0	1.000
*N. pienaari* MZCS 505	in situ	control	14	15	20	93.3		75.0	
S23 31.787		adults	15	15	20	+6.7	1.000	0.0	1.000
E32 34.676		catfish	13	14	20	−0.4	1.000	−5.0	1.000
		tilapia	16	17	20	+0.8	1.000	+10.0	.695

Eggs = number of eggs entering the experiment (N), hatched in 12 hr and in 72 hr; % = hatching rate and pace of the control group, difference from the control in respective treatment groups; *p* = significance of the difference according to Fisher exact test (*p*, significant values in bold), NA = not available. Please note that no effects remained significant when a statistical correction for multiple testing was applied (Benjamini–Hochberg procedure with FDR of 0.05, see Discussion).

Fish predators were represented by wild‐caught *C. gariepinus* and *C. rendalii*. These originated from a temporary pool (S 24° 18.681′; E 33° 1.972′) within the area of distribution of all the killifish species used in the study and were imported to the laboratory in 2015. Although available, we opted not to use lungfish as a test predator because it is well adapted to desiccation (Reichard, 2015); hence, any potential antilungfish strategy involving postponed hatching lacks rationale.

Population‐specific hatching response to predator‐ and conspecific‐conditioned water was tested ex situ (Pinceel et al., 2015). To obtain test water, we separately introduced two adult tilapia (10 and 12 cm total length), a catfish (19 cm total length), and five pairs of adult *N. furzeri* MZCS 414 into three identical 30‐L tanks. Temperature was maintained at 23°C, and the tanks were not filtered but aerated. A fourth tank was prepared (same dimensions and conditions) with water but no fish in order to provide water for a control group. After 4 days of conditioning, water from each tank was filtered through 0.45‐μm laboratory filter (Pinceel et al., 2015). The filtered water was then cooled (Pinceel et al., 2015) to 15°C (Polačik et al., 2016) and immediately used to wet eggs of the three distinct *N. furzeri* populations (38–50 eggs per treatment; Table 1). As a relatively low number of fully developed eggs were available for the *N. furzeri* MZCS 2 population, we trade off the catfish treatment against a higher number of eggs in the remaining treatments. The eggs were group‐wetted in a plastic container using 1 L water per treatment and kept at 25°C for 72 hr. During the time period, all containers were inspected at 12‐hr intervals (a total of six inspections) and the number of hatched fish recorded.

Species‐specific hatching response was tested using a modified methodology due to a negligible response obtained using the ex situ method (see Results). To account for any potential methodological artifact of the ex situ approach, we tested multiple *Nothobranchius* species in situ, thereby more closely reflecting natural hatching conditions. Specifically, 20–50 ready‐to‐hatch eggs of *N. furzeri*,* N. orthonotus*, and *N. pienaari* (Table 1) were placed in a glass container, tightly covered with 0.5‐mm mesh and simultaneously submerged directly in the tank with one of the predators or adult conspecifics of the same population. Hatching temperatures were maintained at 23°C to ensure the welfare of the predators and adult conspecifics (Genade, 2005; Pinceel et al., 2015; Polačik et al., 2016; Prokešová, Drozd, Kouřil, Stejskal, & Matoušek, 2015). Inspections followed the same time schedule as the ex situ method.

### 2.2 Growth experiment

A cohort of *N. furzeri* MZCS 121 fish were raised with olfactory cues from *C. rendalii* either present or absent. We were measuring fish size (total length, from the snout to the end of the tail fin) at regular intervals in order to detect any potential influence of predator kairomone on growth rate. In addition, age at onset of sexual maturity was compared in control and treatment males.

**Figure 1:**
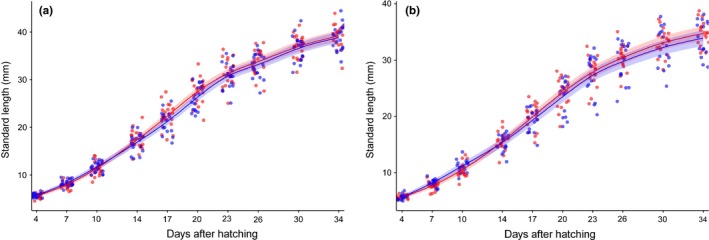
Growth curve of kairomone‐treated (blue) and control (red) *N. furzeri* males (a) and females (b) as predicted by (two‐smoother) generalized additive mixed models. Lines = predicted curve, colored area = 95% confidence interval

Experimental fish originated from a batch of eggs collected after a week of group spawning by 10 males and 20 females. The batch was incubated for 3 months at 25°C and then wetted in a 6‐L tank following a common hatching protocol (Polačik et al., 2016). Hatched juveniles were fed *Artemia* nauplii three times a day. The experiment began when the fish were 4 days old, that is, when they were not too fragile for handling and could be individually housed in a recirculation system. Ninety randomly chosen fish were then digitally photographed, and their total length was measured using ImageJ software (ImageJ, NIH Bethesda, MD) (see Polačik et al., 2014). Measurement continued at 3‐ to 4‐day intervals (Fig. 1) until the end of the experiment (at age 34 days) in order to evaluate growth rate over time (Fig. 1). Immediately after the first body size measurement, the experimental fish were distributed into two identical Aqua Medic Fish Boxes (Aqua Medic GmbH, Germany). Each recirculation system consisted of 45 × 2L tanks with 0.6 L/min water inflow from a filtration unit. Both systems were positioned in the same room, and water temperature was maintained at a constant 28°C, except for brief periods when it dropped by 1–2°C when water was changed (always synchronized in both systems).

Predator kairomone was produced by the same two *C. rendalii* individuals as used in the hatching experiment. We preferred using the cichlids over the catfish due to the tilapia's gape limitation; catfish larger than 15 cm being virtually gape‐unconstrained in terms of *N. furzeri* predation, while tilapiine cichlids are normally limited to juvenile fish (M. Polačik pers. obs.). Potential induction of increased growth rate was more likely using tilapia, therefore, and better supported by general evolutionary theory (e.g., Rodd & Reznick, 1997). Our customized recirculation systems provided enough free space in the filtration unit (40 L) to allow for midterm housing of the tilapia. These were introduced into the treatment recirculation system 4 days before introducing the *N. furzeri* (the filtration unit in the control system was left empty). As *Nothobranchius* spp. live exclusively in stagnant waters (Wildekamp, 2004), constant current from the system's filtration unit would have been stressful to the juveniles, while also effectively compromising feeding on *Artemia* nauplii by washing them away. During the first 6 days of the experiment (i.e., up to the age of 10 days), therefore, kairomones were delivered to the treatment fish on a periodic basis when the filtration unit was turned on for 5 min twice a day (at 9:00 and 16:00), which allowed for a complete change of water in each 2‐L tank. Starting on day 11, the filtration unit was run permanently in both systems.

Experimental *N. furzeri* followed identical feeding regime in both the treatment and control systems. The fish were fed twice a day ad libitum with live *Artemia* nauplii (days 1–6), a mixture of *Artemia* nauplii and chopped frozen bloodworm (days 7–11) and full‐sized frozen bloodworm from day 12 on (Polačik et al., 2016). Uneaten food was siphoned away 15 min after each feeding. To ensure consistent water quality in the treatment and control systems, 40% of the total system water volume (200 L) was changed three times a week. Concentration of NO_2_ and NO_3_ ions was measured before each water change using a commercial test kit (Tetratest NO_2_, Tetratest NO_3_) to ensure that the waste load was the same in both systems. The values recorded remained stable throughout the experiment, with concentration of NO_2_ consistently below detectable values and NO_3_ showing negligible fluctuation between 10 and 20 mg/L (Camargo, Alonso, & Salamanca, 2005).

After the experimental fish had reached maturity, individual housing enabled retrospective sex determination for the juvenile period. Onset of sexual maturity was evaluated in males based on the appearance of first signs of nuptial coloration (Polačik et al., 2014). Mortality and developmental abnormalities such as “belly sliding,” a common occurrence in captive *Nothobranchius* fishes (Genade et al., 2005), reduced the initial 90 experimental fish to 19 males and 15 females in the treatment group and 20 males and 18 females in the control group. Data from any individual that died or showed a defect throughout the experiment were excluded from the analysis.

## 3 Data analysis

### 3.1 Hatching experiment

Separate models were constructed to reveal general trends in (i) population‐specific and (ii) species‐specific hatching response to predator kairomones. Both population‐specific and species‐specific models predicted an effect of two fixed predictors, that is, (iii) *population* or *species* (both categorical factors with three levels) and (iv) *kairomone* (categorical factor with four levels), on a response variable describing the hatching response. Two such response variables were considered: (i) final hatching rate after 72 hr and (ii) hatching pace (i.e., the proportion of eggs hatched after 12 hr to those hatched after 72 hr). As the response variables were binomially distributed (number of successful observations of a fixed number of observations), we used generalized linear models (GLMs) to test for the effect of the predictors.

Additionally, within each of the six replicates (i.e., populations/species, Table 1), the differences between the kairomone treatments and no‐kairomone control in hatching rate and pace were tested using Fisher exact tests. Conducting multiple tests (17 Fisher tests conducted for both hatching rate and hatching pace) increases the probability of committing type I error. Benjamini–Hochberg procedure to control false discovery rate (FDR; Benjamini & Hochberg, 1995) was, therefore, subsequently applied in order to reveal whether the effects found in the Fisher exact tests were attributable to the multiple testing (FDR set to 0.05).

### 3.2 Growth experiment

Kairomone‐treated and control group killifish growth rate was compared using generalized additive mixed models (GAMMs). GAMM was used due to repeated measurements of the same individual, increasing variance with size and nonlinear patterns in fish growth. Single‐smoother models (suggesting no difference in growth curve) and separate smoother models (suggesting the opposite) for treatment and control groups were compared using the Akaike information criterion corrected for finite sample sizes (AICc). Final model validation was performed visually, revealing no pattern in residuals. Separate models were conducted for males and females as their growth rate is known to differ (e.g., Polačik et al., 2014).

Effect of predator kairomone on (i) final total length at the end of the experiment (day 34) and (ii) timing of onset of male nuptial coloration was tested using t tests (separate tests conducted for males and females) and GLM (Poisson distribution corrected for underdispersion, i.e., quasi‐Poisson), respectively. All analyses were performed using R software, version 3.2.4 (R Core Team 2016).

## 4 Results

### 4.1 Hatching experiment

**Figure 2:**
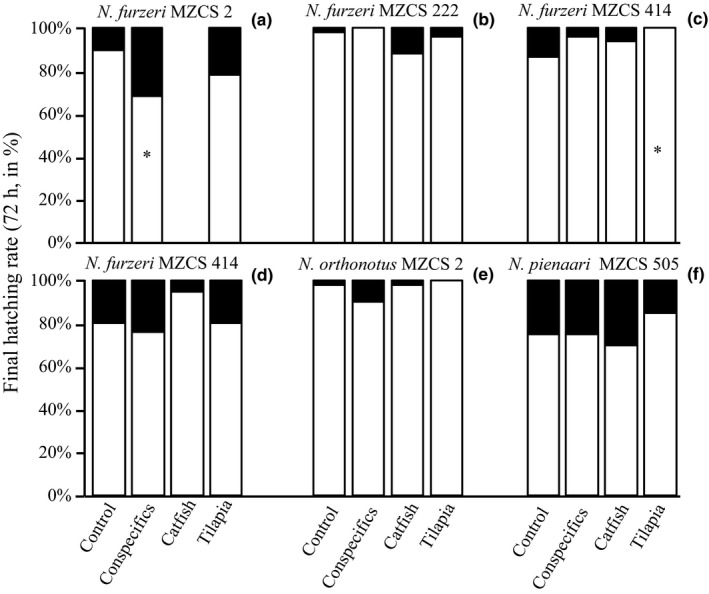
Final hatching rate (72 hr) of kairomone‐treated and control *Nothobranchius* spp. (a–c) = *N. furzeri* populations treated using the ex situ method, (d–f) = *N. furzeri*,* N. orthonotus* and *N. pienaari* treated using the in situ method. White color = the proportion of hatched fish; black color = the proportion of unhatched eggs; * = significant difference from the respective control group

Final (72 hr) hatching rate was relatively high across all treatments, populations, and species, with a mean rate of 87.7% (*SD* = 10.2) and a minimum of 68% (Fig. 2). There was no general trend in the influence of predator‐ or conspecific‐produced kairomones on hatching rate across the populations or species tested (GLM, *df* = 3,7, *p* = .703 for populations, *df* = 3,8, *p* = .193 for species; models without the predictor *kairomone* were more parsimonious than the models with inclusion of the variable: AICc_without_ = 63.9 and AICc_with_ = 82.4 for populations, AICc_without_ = 56.9 and AICc_with_ = 72.8 for species). Within‐replicate tests revealed only a single negative influence on hatching rate of adult conspecifics in *N. furzeri* MZCS 2 population (ex situ method; Fisher exact test, *df* = 1, *p* < .05) (Fig. 2a) and a single positive influence of the *tilapia* treatment in *N. furzeri* 414 population (ex situ method; Fisher exact test, *df* = 1, *p* < .01) (Fig. 2c) (Table 1). None of these two effects were significant when a correction for multiple testing was applied (i.e., Benjamini–Hochberg procedure with FDR of 0.05).

**Figure 3:**
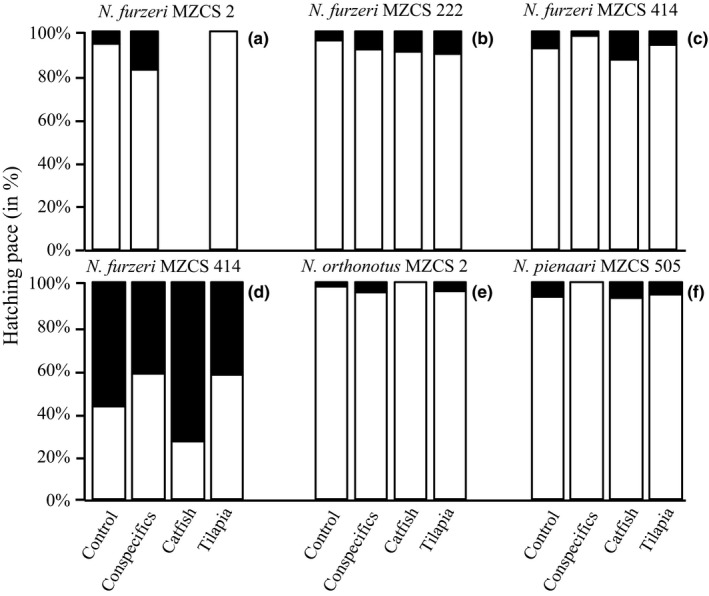
Hatching pace of kairomone‐treated and control *Nothobranchius* spp. (a–c) = *N. furzeri* populations treated using the ex situ method, (d–f)  = *N. furzeri*,* N. orthonotus*, and *N. pienaari* treated using the in situ method. White color = the proportion of eggs hatched after 12 hr to those hatched after 72 hr; black color = the proportion of eggs which did not hatch after 12 hr to those hatched after 72 hr. There was no significant difference from the respective control group in any of the population/species

Pace of hatching was relatively fast. Eggs mainly hatched within the first 12 hr (mean proportion 94% of total hatched eggs, *SD* = 4.5) (Fig. 3). Only in the in situ tested *N. furzeri MZCS* 414 population the mean hatching rate was lower during the first 12 hr and across all treatments (46.1% of the total number of eggs hatched within 72 hr, *SD* = 15.0) (Fig. 3d). Pace of hatching was not influenced by predator‐ or conspecific‐produced kairomones either in general (GLM, *df* = 3,7 and *p* = .438 for populations, *df* = 3,8 and *p* = .695 for species; models without the predictor *kairomone* were more parsimonious than the models with inclusion of the variable: AICc_without_ = 55.2 and AICc_with_ = 75.8 for populations, AICc_without_ = 49.8 and AICc_with_ = 65.8 for species) or within each of the six replicates (Fisher exact tests, all *df* = 1; *p* > .05) (Fig. 3).

### 4.2 Growth experiment

We found no significant effect of tilapia kairomone on growth rate on males (GAMM, *df* = 37, *p* = .461; Fig. 1a) or females (GAMM, *df* = 31, *p* = .537; Fig. 1b). Predator presence did not affect the shape of the growth curve, models with a single smoother providing better results than those with two separate smoothers (males: single‐smoother model, *df* = 8, AICc = 1108.1, two‐smoother model, *df* = 10, AICc = 1122.3; females: single‐smoother model, *df* = 8, AICc = 934.9, two‐smoother model, *df* = 10, AICc = 942.1).

We detected no effect of predator kairomone on age at onset of male nuptial coloration (GLM, *df* = 1,34, *p* = .173). In the absence of the predator, males started to color up at the mean age of 19.1 days (95% CI = 18.19–20.04), while it was 20.05 days in the presence of the predator (95% CI = 19.09–21.04). Similarly, there was no difference in final total length of *N. furzeri* at the end of the experiment (t tests, males: *df* = 1,37, *p* = .672; females: *df* = 1,31, *p* = .409). The mean size of males was 39.19 mm in the absence of the predator (95% CI = 38.00–40.37) and 38.83 mm in the presence of the predator (95% CI = 37.61–40.04). The mean size of females in the absence of the predator was 34.70 mm (95% CI = 33.38–36.01), while it was 33.90 mm (95% CI = 32.46–35.33) in the presence of the predator.

## 5 Discussion

Experiments testing the effect of predator and conspecific kairomones on *Nothobranchius* spp. hatching and growth rate did not support alteration of the killifish's life cycle by chemical cues. Final hatching rates remained high regardless of type of egg treatment, and there was no consistent, treatment‐caused trend observed in the number of eggs hatched or hatching pace in response to predator and conspecific cues over time. Likewise, there was no change in *N. furzeri* growth rate.

To date, hatching rate in response to predator chemical cues has predominantly been studied in invertebrates. These studies have tended to produce mixed results, suggesting a lack of any strong general principle. While dinoflagellates were observed to decrease their excystation rate (Rengefors, Karlsson, & Hansson, 1998), for example, the hatching rate of crustaceans has been shown to either decrease (Lass, Vos, Wolinska, & Spaak, 2005; Spencer & Blaustein, 2001), increase (Lass et al., 2005), or remain unaffected (Waterkeyn et al., 2013). Species‐ and population‐specific sensitivity and reaction to kairomones have been recognized across different taxa (Bernot, Dodds, Quist, & Guy, 2006; Pietrzak et al., 2015; Van Dam & Walton, 2008), most likely reflecting adaptations to local conditions (De Meester, 1996; Waterkeyn et al., 2013). Just one previous study has been undertaken on annual killifish, this showing postponed hatching in Tanzanian *N. steinforti* in reaction to the presence of a nonnative predatory fish (Pinceel et al., 2015). In contrast to our tested *Nothobranchius* spp. distributed in much more arid regions (Mazuze, 2007; Reichard et al., 2014), populations of *N. steinforti* experience two rainy seasons per year and its distribution area is characterized by high interannual rainfall reliability 78.9% (i.e., the mean interannual fluctuation in precipitation is 21.1%; Hamisi, 2013). Such consistent rainfall provides a realistic prospect of further inundations, and under these circumstances, the costs of a missed opportunity could be overbalanced by the benefits (see Introduction; Dayton & Fitzgerald, 2011) and be low enough to allow for evolution of postponed hatching. Nevertheless, contrasting outcome of our experiments and the study of Pinceel et al. (2015) clearly show that this type of antipredator strategy cannot be regarded as a shared capability for the genus *Nothobranchius*.

Three assumptions need to be met to result in evolution of the postponed hatching as an adaptive plastic response. First, the co‐occurrence of the annual fishes and a tilapiine or catfish (i.e., coevolutionary history) must be frequent enough. The colonization rate seems to be relatively high as our field data (unpubl.) from Mozambique over seven rainy seasons yielded 22% of samples from the pools, at least in some years with a documented presence of *Nothobranchiu*s fishes, containing either or both of the two fish predators (tilapiines: 9%; catfish: 8%; both: 5%). The second assumption is that the presence of predators must incur fitness costs to the annual fish. Currently, there are no data available on predation rate of tilapiines and the catfish on *Nothobranchius* spp. in natural habitats, but the predators readily consume adult (catfish) and juvenile (tilapiines) *Nothobranchius* killifish in captivity (M. Polačik unpubl. data), and being extreme food generalists (e.g., Bruton, 1979; Zengeya & Marshall, 2007) their role in *Nothobranchius* spp. predation in the wild is highly plausible. The third assumption is that multiple inundations of the pool occur regularly enough that relying on this future event prevailingly results in a fitness gain. There is evidence that temporary pools sometimes fill repeatedly and may host two or three generations (or cohorts) of annual fish of the tested species within a single rainy season (Polačik et al., 2014; M. Polačik unpubl. data). At the same time, in southern Mozambique the very incidence of the pools, duration of the wet phase, and their repeated filling are subject to great interannual and regional fluctuations (Mazuze, 2007; Blažek et al., 2013; Terzibasi et al. 2013, Reichard et al., 2014; Vrtílek and Reichard 2016, Blažek et al. 2017). The relative benefits of avoiding fish predators are likely to be overbalanced by multilevel uncertainty of inundations. In addition, survival of individuals that postponed their hatching is by no means guaranteed should a second inundation arrive as predators could still reinvade the system. Summing up, while the first two assumptions seem to be fulfilled in the examined species, the third is not as the irregularities in the rainfall pattern present a strong barrier for the evolution of the adaptive response.

While a significant effect of predator or conspecific kairomones was found in two replicates of the 17 noncontrols (Table 1, Fig. 2), we believe that the significance can be attributed to the multiple testing (type I error; no effect when correction for multiple testing applied). Our view to treat the two effects as coincidental comes from (i) their inconsistency across populations and species, (ii) the difficulty of accommodating them within coevolutionary and ecological contexts if we attempt to explain them as local adaptations. Specifically, we observed a significantly decreased hatching rate in the presence of adult conspecifics (*N. furzeri* MZCS 2 population; Fig. 2a) and a significantly increased hatching rate in the presence of redbreast tilapia (*N furzeri* MCZS 414 population; Fig. 2c). First, particular populations may not have a coevolutionary history with a specific predator, and these are unlikely to evolve an effective antipredator strategy (Rieder, Newbold, Sato, Yasuda, & Evans, 2008). In contrast, eggs of all annual killifish will share the same habitat with conspecific adults for some time. Thus, any local variation in sensitivity to conspecific kairomones is highly implausible. Inhibitory factors produced by adults will have acted on embryos before the prehatching developmental stage (Inglima et al., 1981), making any additional functional control of hatching largely redundant. Second, we see no adaptive explanation for the increase in hatching rate in the presence of a predator (Lass et al., 2005). Therefore, we also view the significantly higher hatching rate in *N. furzeri* MZCS 414 in the presence of the tilapia kairomone as a coincidence caused by a relatively low hatching rate in kairomone‐free control (Fig. 2c).

Hatching pace in the *N. furzeri* MZCS 414 in situ replicate was considerably slower than for the same population hatched using the ex situ method, as well as for all other populations and species (Table 1, Fig. 3d). Protracted hatching over time within a single inundation is believed to represent a bet‐hedging strategy whereby late hatchers benefit from factors such as increased food availability (Vanoverbeke & De Meester, 2009; Waterkeyn et al., 2013). At the same time, they may also suffer from increased predator density as a consequence of decreasing water level (Touchon, Gomez‐Mestre, & Warkentin, 2006). In our experiment, fish of the same population hatched faster using the ex situ method, suggesting either an environmental trigger decelerating hatching pace in the in situ replicate or a potential stochastic component in the hatching speed of *N. furzeri*. The in situ replicates used warmer water (23°C vs 15°C) and higher water levels (35 vs 5 cm) than the ex situ replicates (see Methods), and these factors are likely to have interacted. Both water temperature (Genade, 2005; Polačik et al., 2016) and hydrostatic pressure (Genade, 2005) have been reported as influencing annual killifish hatching, perhaps serving as an abiotic cue informing on the hatching environment (Vanoverbeke & De Meester, 2009). Notably, *N. orthonotus* and *N. pienaari,* both of which were hatched using the same in situ method, did not react by decelerating their hatching speed, suggesting either that sensitivity to these types of environmental cue is species‐ or population‐specific or that there is a stochastic component involved, adding to the generally “bet‐hedged” lifestyle of annual killifish (Furness, Lee, & Reznick, 2015; Pinceel et al., 2015; Polačik & Podrabsky, 2015; Polačik et al., 2014).

### 5.1 Growth experiment

The presence of redbreast tilapia kairomone in the water did not induce a shift in growth rate in *N. furzeri* (Fig. 1); consequently, maturation rate and final total length in the treatment and control groups did not differ.

In many animal taxa, growth rate modulation in response to predatory cues appears to follow specific, efficient modes of predator avoidance. Small planktonic crustaceans and insects typically tend to decrease predator‐encounter rate by slowing down growth (Dawidowicz & Wielanier, 2004; Gliwicz & Maszczyk, 2007; Pijanowska, Dawidowicz, Howe, & Weider, 2006), perhaps as a consequence of reduced foraging activity (Jourdan et al., 2016). In contrast, size reduction is less common in vertebrates (Barry, 2014; Smith, Burgett, Temple, & Sparks, 2016) as they can more easily escape predation by growing into a size refuge (e.g., through exceeding the predator's prey size range or through metamorphosis). As a result, growth is more often accelerated in vertebrates in the presence of predators (Costa & Kishida, 2015; Mogali, Saidapur, & Shanbhag, 2016). We did not expect to see an increased growth rate in kairomone‐treated *N. furzeri* despite the fact that the temporary pools in their range are typically invaded by gape‐limited, juvenile (up to 4 cm total length) tilapiine cichlids (M. Polačik, unpubl. data). In our system, the presence of a predator that is effectively preying mainly on juvenile stages selects in the same way as habitat seasonality, that is, toward rapid growth. The tested species *N. furzeri* is the fastest maturing vertebrate in the world (Blažek et al., 2013), characterized by a tight linkage between body size and sexual maturation (Blažek et al., 2013; Polačik et al., 2014). As a result, the fish appear to grow at their maximum rate with no spare capacity for any additional acceleration, at least in captivity when fed *Artemia* nauplii and chironomid larvae. Alternatively, it is possible that coevolution with the predator has been too loose to induce any specific antipredator strategy. While we specifically chose a *N. furzeri* population originating from a pool with documented occurrence of tilapiines (M. Polačik, unpubl. data), we only have data on the tilapiine–killifish association over several generations (see above), and the long‐term frequency of cichlid invasions is unknown. Life‐history adaptation can only evolve if the strength of induction is sufficient and costs are lower than benefits in terms of a fitness gain (Magurran, 2005).

Male *N. furzeri* in the treatment and control groups started to color up at the same age in accordance with the lack of difference in growth rate (Fig. 1). *Nothobranchius* spp. are “income breeders,” that is, fish that store eggs for only brief periods and spawn continuously (Wooton & Smith, 2015). In contrast to seasonal bout spawners, income breeders gain relatively few fecundity benefits by postponing reproduction to a period when a larger body size is achieved. Furthermore, rapid reproduction is more important in an unpredictable environment than any prospect of increased fecundity in the future (Blažek et al., 2013). A within‐cohort difference in *N. furzeri* maturation rate was documented in the study of Polačik et al. (2014), although this was accompanied by dissimilarity in growth rate and body size. *Nothobranchius* fishes reach sexual maturity as soon as they reach the minimum size physiologically suitable for reproduction. Based on the evidence now accumulated, it would appear plausible that a difference in growth rate is a necessary prerequisite for any distinction in intrapopulation maturation rate in *Nothobranchius* spp.

## 6 Conclusions

We found no evidence that Mozambican annual killifish of the genus *Nothobranchius* have evolved an antipredator strategy involving hatching rate modulation and no support for their ability to modulate growth rate in response to predator kairomones. The costs of a missed opportunity can be very high in unpredictable ecosystems, including total reproductive failure. In regions with extremely erratic rainfall, the evolutionary role of fish predation appears to be inferior to that of environmental unpredictability, preventing induction of the antipredator adaptations tested in this study. To further clarify the role of local environmental conditions, we suggest that future studies on the extent of costly antipredator adaptations in annual fish should include tests using replicated species or populations from regions with contrasting rainfall predictability and focus also on the more plastic traits such as behavioral activity.

## Conflict of Interest

None declared.
